# Outcome of lung cancer surgery and proportion of lung cancer patients eligible for surgery in five Finnish hospitals in 2018, real world study

**DOI:** 10.2340/1651-226X.2025.43398

**Published:** 2025-07-23

**Authors:** Ville Paappanen, Jenny Tikka, Arja Jukkola, Tuula Klaavuniemi, Liisa Sailas, Maria Tengström, Hanne Kuitunen, Teemu Riekkinen, Satu Tiainen, Outi Kuittinen

**Affiliations:** a Faculty of Health Medicine, Institute of Clinical Medicine, University of Eastern Finland, Kuopio, Finland; b Department of Oncology, Tampere University Hospital, Tampere, Finland; c Department of Oncology, Joensuu Central Hospital, Joensuu, Finland; d Cancer Center, Kuopio University Hospital, Kuopio, Finland; e Department of Oncology, Oulu University Hospital, Oulu, Finland; f Department of Surgery, Kuopio University Hospital, Kuopio, Finland; g FICAN East, the Wellbeing Services, county of North Savo, Kuopio, Finland

**Keywords:** Lung cancer, surgery, operability, survival, limited stage

## Abstract

**Background and purpose:**

Lung cancer is one of the most common malignancies worldwide and is associated with high mortality. In Finland, overall lung cancer survival is lower than in other Nordic countries. A recent Finnish Cancer Registry publication reported that only 11.8% of patients underwent surgery. We aimed to assess whether operability and surgical outcomes contribute to Finland’s inferior lung cancer survival rates.

**Material and methods:**

This retrospective study analysed patient data of five Finnish hospital databases. We focused on potentially operable patients, specifically those with non-small cell lung cancer in stage I–IIIA according to computer tomography. A total of 156 patients met the staging criteria, of whom 77 underwent surgery. Among potentially operable patients, 50.6% were deemed ineligible for surgery due to various factors, including poor pulmonary or cardiac function, comorbidities, or localised tumour spread.

**Results:**

In our material 156 out of 545 were potentially operable and 77 were operated. 2-year overall survival for operated patients was 79%.

**Interpretation:**

We found that patients with lung cancer in Finland present with poorer overall health, a slightly more advanced stage distribution among potentially operable cases, and a lower overall rate of surgical treatment compared to other Nordic countries. Additionally, patients in Finland tend to undergo surgery at more advanced stages. These factors likely contribute to Finland’s lower lung cancer survival rates. This study underscores that delayed diagnosis and a lower proportion of patients undergoing surgery may be key contributors to Finland’s poorer treatment outcomes.

## Introduction

Lung cancer (LC) was the second most commonly diagnosed cancer and was the leading cause of cancer deaths worldwide in 2020, accounting for 11 and 18% of all new cancer cases and cancer deaths, respectively [1]. Globally, the majority of LC cases in 2020 were non-small cell lung cancer (NSCLC), comprising 72 and 75% of cases in men and women, respectively. Small cell lung cancer (SCLC) accounted for 11 and 9% of male and female LC cases, respectively [2]. SCLC is often diagnosed at an advanced stage, making curative treatment unfeasible, whereas stage I–III NSCLC can often be treated with curative intent. In metastatic NSCLC (stage IV), the treatment goal is to slow disease progression and alleviate symptoms.

NSCLC treatment options include surgery, radiotherapy (RT), chemoradiotherapy, chemotherapy, immunotherapy (IO), and targeted therapies. Curative treatments primarily involve surgery, with or without (neo)adjuvant therapy, or RT. For locally advanced disease, chemoradiation is also an option, with surgery remaining the gold standard. However, most NSCLC cases are diagnosed at an advanced, inoperable stage [3].

In the Nordic countries, the 5-year relative survival for males with LC was approximately 10% between 1998 and 2002, based on NORDCAN data [4, 5]. Since then, survival rates have significantly improved, reaching 26.8% in Sweden, 28.2% in Norway, and 26.2% in Denmark between 2018 and 2022. However, Finland has lagged behind, with a 5-year survival rate of only 16.7% in men and 27.3% in women [4]. The underlying causes of this survival gap between Finland and other Nordic countries remain unclear due to a lack of registry data detailing patient characteristics, disease staging, and treatment patterns.

A recent national report on LC in Finland revealed regional differences in treatment approaches. The report indicated that patients in Northern and Eastern Finland are more frequently treated with RT or pharmacologic therapies, while surgical intervention remains less common. According to the report, approximately 10% of Finnish patients with LC underwent surgery, while fewer than one-third received RT, and approximately 20% received drug therapy [6]. As this report represents an initial draft of a national cancer quality registry, concerns remain regarding data quality and completeness.

We aimed to provide a detailed analysis of the characteristics and treatment patterns of Finnish patients with LC across five hospitals to better understand potential reasons for their poor survival outcomes. We focused particularly on surgical treatment, as it offers the highest curative potential. Our analysis examined the proportion of newly diagnosed patients with LC who underwent surgery, their surgical outcomes, and the reasons for ineligibility for operative treatment in a comprehensive cohort of patients diagnosed with LC in Finland during 2018.

## Materials and methods

### Study population

The study population comprised 545 patients aged ≥18 years who were diagnosed with any type of LC, ICD 10 code C34, in 2018 across three Finnish university hospitals and two central hospitals; Kuopio University Hospital, Tampere University Hospital, Oulu University Hospital, Mikkeli Central Hospital, and North Karelia Central Hospital. For this substudy, we selected patients potentially eligible for surgical treatment based on the following criteria: diagnosis of NSCLC (*n* = 395) and staging between I and IIIA based on computed tomography (CT) scans. Based on these criteria, the final study cohort consisted of 156 potentially operable NSCLC patients.

**Figure 1:**
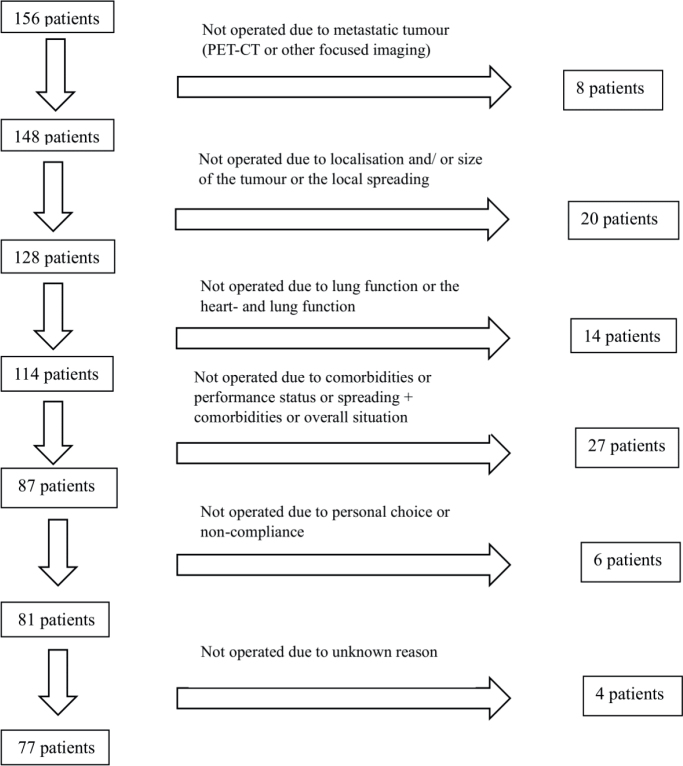
Reasons for withdrawal from operation.

NSCLC diagnoses were confirmed using CT scans and histopathological or cytopathological analyses. Samples were obtained via fine- or coarse-needle biopsies, and in some cases, through sputum or pleural fluid aspiration. Needle biopsies were performed transthoracically, bronchoscopically, or surgically using thoracoscopy or thoracotomy. In some cases, subsequent imaging and reassessment led to a change in staging, which resulted in the withdrawal of patients from surgical consideration (Figure 1). A delay from time of diagnosis to the time of treatment could also have an impact on this. The median time from diagnosis to treatment for operative patients was 69 days (interquartile range [IQR] 53-92) and for non-operative patients 65 days (IQR 46–116). The diagnostic process and treatment selection included physical examinations and laboratory testing. Lung function was assessed using pulmonary function tests when feasible.

Permission to use patient data for this study was granted by The Finnish Social and Health Data Permit Authority, Findata. According to Finnish law, data concerning fewer than five patients cannot be published. Therefore, any instance involving fewer than five patients is indicated accordingly, along with associated percentages.

### Patient and treatment characteristics

Patient characteristics included age, sex, and medical history. Medical history encompassed comorbidities, smoking status, laboratory test results, pulmonary function tests, and other health assessments, tumour characteristics, and current medications. Patients were evaluated using the Charlson Comorbidity Index (CCI), age-adjusted-CCI (AACCI), and World Health Organization (WHO) performance status (PS) scale, which is equivalent to ECOG PS scale [7]. The CCI score was calculated based on patient comorbidities, with higher scores indicating greater comorbidity burden. Comorbidities assessed included for example cardiovascular diseases, liver and kidney diseases, diabetes, chronic pulmonary diseases and prior cancer diagnoses [8]. The AACCI extends the CCI by incorporating additional risk points based on patients’ age [9]. Smoking status was classified as current smoker, ex-smoker, or never-smoker. Tumour characteristics included histopathological diagnosis, mutational status, and tumour-node-metastasis (TNM) classification according to the 8th Edition of the TNM Classification System. For patients who underwent surgery, pathological TNM (pTNM) classification was confirmed. First-line treatment was categorised into the following groups: surgery; chemoradiotherapy; RT with curative intent, including conventional RT and stereotactic body radiotherapy (SBRT); chemotherapy; targeted agent drug therapy; IO; and best supportive care (BSC). Palliative RT administered for symptom control was included in BSC. Due to the small sample size (fewer than five patients), IO and targeted drug therapy were combined in statistical analyses. Detailed patient level data were collected in 2021. Survival status was updated from national registries 1.5.2022.

### Statistical analyses

Data were analysed using IBM SPSS Statistics 29, the R programming language, and RStudio IDE. Pearson’s chi-squared test was used to assess differences in the distributions of categorical variables between treatment groups. To evaluate differences between CCI and AACCI score groups, both the Wilcoxon rank-sum and Pearson’s chi-squared tests were applied. To assess the association between comorbidity indices and treatment, logistic regression analyses were performed. Model coefficients were exponentiated to obtain odds ratios (ORs) with corresponding 95% confidence intervals (CIs). Statistical significance was set at *p* < 0.05. Overall survival (OS) was analysed using Kaplan–Meier survival curves. OS was defined as the duration of time from the date of the start of the therapy to the date of death from any cause and was censored if patient was alive at the date of last follow-up (1.5.2022). The log-rank test was used to assess statistical significance in survival analyses.

## Results

### Characteristics of operated and non-operated patients

The characteristics of patients are presented in Table 1. Of the 156 stage I-IIIA potentially operable NSCLC patients, 49.4% (*n* = 77) underwent surgery, while 50.6% (*n* = 79) did not. The proportion of female patients was higher among the operated group compared to the non-operated group, 46.8% (*n* = 36) vs. 26.6% (*n* = 21), respectively (*p* = 0.014). Additionally, a greater proportion of the operated patients were younger (*p* = 0.002) and had a better PS (*p* = 0.001), with 94.7% (*n* = 72) of the operated and 65.8% (n = 50) of the non-operated patients classified as WHO 0–1. Patients who underwent surgery had LC at an earlier stage compared to non-operated patients (*p* = 0.001). Among the two most common histological subtypes of NSCLC, adenocarcinoma was more prevalent among operated patients than non-operated patients (61.0% [*n* = 47] vs. 36.4% [*n* = 28], *p* = 0.003). Conversely, squamous cell carcinoma was less common in the operated group (31.2% [*n* = 24]) compared to the non-operated group (51.9% [*n* = 40]).

**Table 1:** Patients’ characteristics.

Baseline patient characteristics					
Variable	*N*	Overall, *N* = 156^1^	Non-operative, *N* = 79^1^	Operative, *N* = 77^1^	*p* ^2^
**Age at diagnosis**	156				0.002
< 61		19/156 (12.2%)	7/79 (8.9%)	12/77 (15.6%)	
61–70		55/156 (35.3%)	23/79 (29.1%)	32/77 (41.6%)	
71–80		59/156 (37.8%)	29/79 (36.7%)	30/77 (39.0%)	
> 80		23/156 (14.7%)	20/79 (25.3%)	< 5/77 (< 6.5%)	
**Sex**	156				0.014
Male		99/156 (63.5%)	58/79 (73.4%)	41/77 (53.2%)	
Female		57/156 (36.5%)	21/79 (26.6%)	36/77 (46.8%)	
**WHO score**	152				< 0.001
WHO 0		66/152 (43.4%)	19/76 (25.0%)	47/76 (61.8%)	
WHO 1		56/152 (36.8%)	31/76 (40.8%)	25/76 (32.9%)	
WHO 2		17/152 (11.2%)	14/76 (18.4%)	< 5/76 (< 6.6%)	
WHO 3		12/152 (7.9%)	11/76 (14.5%)	< 5/76 (< 6.6%)	
WHO 4		< 5/152 (3.3%)	< 5/76 (6.6%)	0/76 (0.0%)	
Unknown		4	3	1	
**Smoking status**	151				0.3
Current Smoker		47/151 (31.1%)	28/76 (36.8%)	19/75 (25.3%)	
Ex-Smoker		78/151 (51.7%)	37/76 (48.7%)	41/75 (54.7%)	
Never-Smoker		26/151 (17.2%)	11/76 (14.5%)	15/75 (20.0%)	
Unknown		5	3	2	
**Histology**	154				0.003
SCC		64/154 (41.6%)	40/77 (51.9%)	24/77 (31.2%)	
Adenocarcinoma		75/154 (48.7%)	28/77 (36.4%)	47/77 (61.0%)	
Carcinoid		5/154 (3.2%)	< 5/77 (< 6.5%)	< 5/77 (< 6.5%)	
Other		10/154 (6.5%)	8/77 (10.4%)	< 5/77 (< 6.5%)	
Unknown		2	2	0	
**cTNM stage**	156				< 0.001
IA		61/156 (39.1%)	22/79 (27.8%)	39/77 (50.6%)	
IB		17/156 (10.9%)	8/79 (10.1%)	9/77 (11.7%)	
IIA		7/156 (4.5%)	< 5/79 (< 6.3%)	< 5/77 (< 6.5%)	
IIB		28/156 (17.9%)	12/79 (15.2%)	16/77 (20.8%)	
IIIA		43/156 (27.6%)	34/79 (43.0%)	9/77 (11.7%)	

WHO: World Health Organization.

1 *n/N* (%).

2 Pearson’s Chi-squared test.

The CCI scores of the patient population are presented in Table 2. Operated patients had significantly lower CCI scores, indicating fewer comorbidities, compared to non-operated patients (*p* = 0.025). The proportion of patients with a CCI score of 0 was higher among the operated group (23.7% [*n* = 18]) than among the non-operated group (11.5% [*n* = 9]). Logistic regression analysis, with coefficients exponentiated to obtain ORs, showed that both CCI and age-adjusted CCI were significantly associated with the likelihood of undergoing surgery **(**CCI: OR = 0.81, 95% CI: 0.68–0.96, *p* = 0.021; Age-Adjusted CCI: OR = 0.81, 95% CI: 0.67–0.96, *p* = 0.018). This suggests that higher scores decreased the likelihood of the event, indicating that for each one-point increase in CCI score, the probability of receiving surgical treatment decreased by 19% (*p* = 0.021) (Table 2).

**Table 2:** Patients’ characteristics: Charlson Comorbidity Index.

Baseline comorbidity (CCI and age-adjusted CCI) by treatment group					
Variable	*N*	Overall, *N* = 156^1^	Non-operative, *N* = 79^1^	Operative, *N* = 77^1^	*p* ^2,3^
**CCI**	154	2.18 (1.97)	2.55 (2.15)	1.80 (1.71)	0.021
Unknown		2	1	1	
**CCI categorical**	154				0.025
0		27/154 (17.5%)	9/78 (11.5%)	18/76 (23.7%)	
1		40/154 (26.0%)	20/78 (25.6%)	20/76 (26.3%)	
2		39/154 (25.3%)	19/78 (24.4%)	20/76 (26.3%)	
≥ 3		48/154 (31.2%)	30/78 (38.5%)	18/76 (23.7%)	
Unknown		2	1	1	
**Age-adjusted CCI**	154	4.17 (1.99)	4.55 (2.15)	3.78 (1.74)	0.019
Unknown		2	1	1	
**Age-adjusted CCI categorical**	154				0.043
1		< 5/154 (< 3.2%)	0/78 (0.0%)	< 5/76 (< 6.6%)	
2		25/154 (16.2%)	9/78 (11.5%)	16/76 (21.1%)	
≥ 3		127/154 (82.5%)	69/78 (88.5%)	58/76 (76.3%)	
Unknown		2	1	1	

1 Mean (SD); n/N (%).

2 Wilcoxon rank sum test.

3 Pearson’s Chi-squared test.

### Treatment characteristics of operated patients

Among the operated patients (*n* = 77), lobectomy was the most common procedure, performed in 60 cases (77.9%). Bilobectomy was conducted in 6 patients (7.8%), and sublobar resection in 7 patients (9.1%). Pneumectomy was performed in < 5 patients. Surgery was performed using video-assisted thoracoscopic surgery (VATS) in 44 patients (58.7%), and via thoracotomy in 24 patients (32.0%). In 7 cases (9.3%) VATS procedures were converted to thoracotomy intraoperatively. Surgical method data were missing for 2 patients.

Postoperative complications occurred in 22.1% (*n* = 17) of the operated patients. Of these, 6 patients developed postoperative pneumonia. Other complications included arrhythmia requiring treatment, pulmonary embolism, emphysema, severe pneumothorax, empyema, atelectasis requiring bronchoscopy, and surgical site infections such as cellulitis.

### Treatment characteristics of non-operated patients

A total of 79 patients (50.6%) did not undergo surgery. The reasons for withdrawal from surgical treatment are presented in Figure 1. Among the initially considered operable patients, 28 (17.9%) were not operated on due to findings from additional staging: 8 patients had metastatic disease detected via PET-CT or other focused imaging and 20 patients had locally advanced disease, large tumour size, or tumours in challenging locations. Furthermore, 14 patients (9.0%) were deemed ineligible for surgery due to poor lung and/or heart function, and 27 patients (17.3%) were not operated due to a combination of comorbidities, decreased PS, or disease progression combined with comorbidities. Moreover, 6 patients (3.8%) declined surgery due to personal choice or non-compliance. The reason for non-operation remains unknown for 4 patients (2.6%).

Of the non-operated patients, 28 (35.4%) received BSC, including palliative RT, while 51 (64.6%) underwent other treatments. The treatments administered to non-operated patients are presented in Table S1. Curative-intent RT was administered to 29.1% (*n* = 23) of non-operated patients, while 16.5% (*n* = 13) received chemoradiotherapy (with cisplatin-etoposide or cisplatin). Chemotherapy, was administered to 10 (12.7%) patients. IO-treatment or targeted drug therapy was also administered to 5 (6.3%) patients.

## Survival

Among the operated patients, a total of 25 (32.5%) had died by May 1, 2022, while 52 (67.5%) remained alive. Data on the cause of death were reliably available until December 31, 2020, and based on this information, 18 patients (23.4% of all operated patients) died from LC. A total of 90-days post-operation mortality was < 5 patients. In contrast, among the non-operated patients, 62 (78.5%) had died by May 1, 2022, while 17 (21.5%) remained alive. Based on data until December 31, 2020, the number of patients dead from LC was 44 (55.7%) among the non-operated patients. Patients who remained alive at the end of the follow-up were either in remission or living with cancer. Median follow-up time was 32 months.

**Figure 2:**
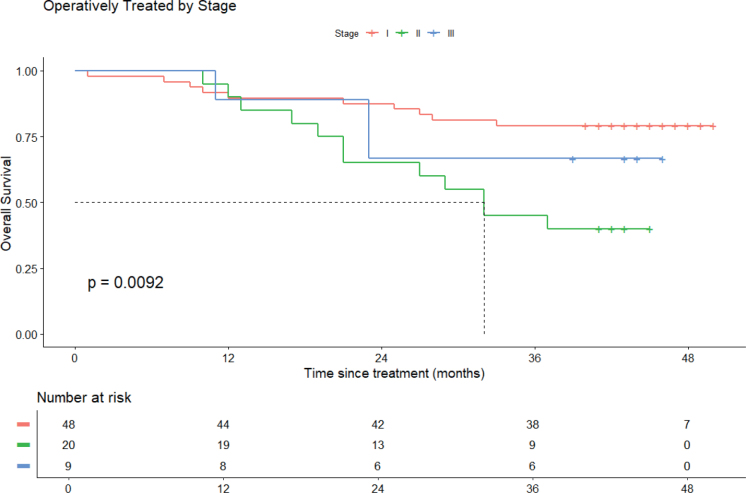
Overall survival of operated patients according to stage.

Survival outcomes for operated patients, stratified by stage, are presented in Figure 2, while survival outcomes for non-operated patients by stage are shown in Figure S1. Stage I patients exhibited the highest OS rates among both operated and non-operated groups. The survival rates for both operated and non-operated patient groups are provided in Table 3. For operated patients across all stages, the 2-year OS rate was 79%, while the 3-year OS rate was 69%. The 2- and 3-year-survival rates stratified by stage for both groups are provided in Table S2.

**Table 3:** Two- and three-year survival rates of operated and non-operated patients.

Survival outcomes at 24 and 36 months by operative versus non-operative treatment			
Characteristic	*N*	24 Month (95% CI)	36 Month (95% CI)
**Overall survival**	156		
Non-operative		34% (24, 45)	28% (18, 38)
Operative		79% (68, 87)	69% (57, 78)

The survival of non-operated patients, based on their treatment modality, is illustrated in Figure S2, with corresponding survival rates in Table 4. Figure S2 compares OS among non-operated patients receiving curative-intent radiation (SBRT or conventional RT), chemoradiotherapy, and palliative treatment. The highest survival rates were observed in patients who received SBRT, with a 2-year OS of 53%. In contrast, among patients who did not receive curative-intent RT nor chemoradiotherapy, the 2-years OS was 23%.

**Table 4:** Survival rate of non-operated patients according to treatment.

Survival outcomes at 24 and 36 months by radiotherapy type for non-operatively treated patients			
Characteristic	*N*	24 month (95% CI)	36 month (95% CI)
**Overall survival**	79		
Chemoradiotherapy	13	38% (14, 63)	31% (9.5, 55)
SBRT	17	53% (28, 73)	47% (23, 68)
Conventional RT	6	50% (11, 80)	33% (4.6, 68)
No radiotherapy	43	23% (12, 37)	19% (8.7, 31)

RT: radiotherapy; SBRT: stereotactic body radiotherapy.

## Discussion

To investigate potential reasons for the poor outcome of Finnish patients with LC, we collected comprehensive clinical data of NSCLC patients diagnosed in Finland in 2018, with a particular focus on operative treatment, which is considered to have the highest curative potential. In our study population there were 156 potentially operable patients (stage I–IIIA NSCLC), out of which 77 were eventually operated. The 2-year OS for operated patients was 79%. Our findings suggest that fewer patients in Finland were diagnosed in potentially operable stages (I–IIIA), a smaller proportion of those with potentially operable disease underwent surgery, and Finnish patients had more comorbidities and poorer PS compared to their counterparts in other Nordic countries.

Despite the similarities in healthcare systems, LC outcomes in Finland have fallen behind those in other Nordic countries [4]. Between 1998 and 2002, the 5-year-survival rates for male LC patients were relatively similar across the Nordic countries: Finland 9.7%, Sweden 11.8%, Norway 10.0%, Denmark 8.9%, and Iceland 11.9%. However, while outcomes have improved in other Nordic countries, Finland has lagged behind. The 5-year survival rate for male patients with LC between 2018 and 2022 was 16.7% in Finland, compared to 26.8% in Sweden, 28.2% in Norway, 26.2% in Denmark, and 28.4% in Iceland [4]. Similar trends were observed among female patient population, although the prognosis of female LC patients is better.

Due to the lack of a national LC registry providing detailed data on patient, disease, and treatment characteristics in Finland, we retrospectively collected patient data from five hospitals to gain further insight into potential causes of inferior survival outcomes. It should be noted that this study is not fully population-based, as three of these hospitals (Kuopio, Oulu, and Tampere) are referral centres for potentially operable patients. Comparative data on LC statistics in other Nordic countries were sourced from the Norwegian Lung Cancer Report [10], the 2019 Iceland Lung Cancer Report [11], the 2024 study by Oskardottir et al. for Sweden [12], and the 2022 study by Ehrenstein et al. for Denmark [13].

In our Finnish NSCLC cohort from 2018, 39.5% of patients were diagnosed in stage I-IIIA (19.7% stage I, 8.9% stage II, 10.9% stage IIIA), which is considered potentially suitable for curative-intent treatment [14]. In comparison, the corresponding percentages were 48.7% (30.6% stage I, 8.8% stage II, 9.4% stage IIIA) in Norway (2018–2022) [10], 50.4% (25.8% stage I, 6.6% stage II, 18.0% stage III) in Sweden (2020–2022) [12], 41.0% (21.0% stage I, 8.0% stage II, 12.0% stage III) in Iceland [11], and 44.0% (stage-specific breakdown not available) in Denmark (2013–2018) [13]. Notably, Finland had a lower proportion of stage I patients, that is those with the best prognosis, compared to Norway and Sweden (19.7% vs. 30.6 and 25.8%, respectively). However, the Norwegian and Swedish data are from slightly more recent years, and our Finnish dataset may be slightly overestimated as it includes referral centre data, encompassing potentially operable patients from non-surgical hospitals.

Among Finnish NSCLC patients diagnosed in 2018, 49.4% (77/156) of those in initially staged as having stage I–IIIA underwent surgery. In Norway in 2018–2022 54.5% of stage I–IIIA LC patients underwent surgery (including some SCLC patients) [10] and the corresponding percentage in Denmark in 2013–2018 was 59.2% [13]. In our cohort, patients were excluded from surgery for various reasons, including comorbidities, decreased PS, metastatic disease found during further staging, impaired lung or heart function, tumour localisation, or compliance issues. Given the retrospective nature of this study, we cannot definitively determine whether clinical practice differences contributed to the lower surgical rates in Finland compared to other Nordic countries.

Our findings suggest that in Finland, fewer LC patients are diagnosed at potentially operable stages and even smaller proportion of those eligible for surgery ultimately undergo the procedure. In our NSCLC cohort, 19.5% of all patients (all stages) underwent surgery, compared to 11.8% in the population-based Finnish Cancer Registry [15], 26.6% in Norway (including SCLC) [10], 26.0% in Denmark [13].

Furthermore, post-operative survival outcomes among Finnish LC patients appear slightly worse than those in other Nordic countries. The 2-year OS rate among Finnish NSCLC patients who underwent surgery was 79%, while the 3-year OS rate was 69% in 2018. In contrast, Norway reported a 5-year-survival rate of 76.2% in 2022 [10], surpassing the 3-year survival rate of our Finnish cohort. Despite relatively similar stage distribution among the operated patients across Nordic countries, (62.3% of Finnish surgical patients had stage I disease compared to 65.2% in Norway [including SCLC] in 2018 [10] and 71.0% in Sweden [12]), postoperative survival remained lower in Finland.

One potential factor contributing to this discrepancy is the overall poorer health status and higher comorbidity burden among the Finnish LC patients. In our cohort, 17.5% of Finnish stage I-IIIA NSCLC patients had a CCI score of 0, 51.3% had a score of 1–2, and 31.2% had a score of 3 or higher. In contrast, Denmark reported 39.6% of patients with a CCI score of 0, 40.4% with a score of 1–2, and 20.0% with a score of 3 or higher [13]. Similarly, PS was worse among Finnish patients. The proportion of potentially operable patients with WHO/ECOG 0 was 43.4% in our Finnish cohort, compared to 39.3% in Norway (2018–2022, including stage I-III SCLC) [10], 52.7% in Denmark (2013–2018) [13], and 42.0% in Sweden (2008–2019) [12]. Patients with WHO/ECOG 1–2 accounted for 48.0% in Finland, while Norway reported 52.6% (including stage I-III SCLC) [10], Denmark 42.8% [13], and Sweden 53.2% [12]. The percentage of patients with WHO/ ECOG 3 or higher was 8.6–11.2% in Finland, 8.1% in Norway (including stage I-III SCLC) [10], 4.5% in Denmark [13], and 4.8% in Sweden [12].

In general the reason for the better survival of women is considered to be due to them being diagnosed at an earlier stage, them being in better condition and generally younger. Among female patients there are more non-smokers and their LC histology is more commonly adenocarcinoma, which has better treatment results compared to squamous cell carcinoma [16]. These differences could have an impact on females being operated more frequently as seen in Table 1. Our finding of gender differences in this study’s LC patient population is similar to other studies [16].

Squamous cell carcinoma was more prevalent in our Finnish patient cohort (41.6% of stage I-IIIA NSCLC) than in Norway (26.4% in 2018–2022) [10] and Sweden (28.0% in 2008–2019) [12]. However this does not explain the survival disparities as the trend of LC survival in Finland compared to other Nordic countries was similar between different histological subtypes [5].

A key strength of this study is its multi-institutional setting. According to the Finnish Cancer Registry, 2,927 new lung and tracheal cancer cases were diagnosed in Finland in 2018 (1,107 among females and 1,820 among males) [17]. Our study’s patient population (*n* = 545) represents 18.6% of all Finnish LC cases from that year, providing a substantial dataset for analysis. The Finnish Cancer Registry includes all LC diagnoses; those diagnosed post mortem and those who have not been sent to hospital. Additionally, the detailed, manually collected hospital record data enhances the reliability of our findings.

However, the study also has limitations. The relatively small sample size is a constraint, as the study includes only patients diagnosed in 2018. Furthermore, cross-country comparisons are based on aggregate data rather than individual patient-level analyses, and the availability of comparable public data varies between countries.

## Conclusion

Our study identifies several factors likely contributing to the inferior outcomes of Finnish LC patients. These include a lower proportion of early-stage diagnoses, and a generally poorer health status among potentially operable patients, as indicated by CCI scores and WHO PS. These factors lead to a lower proportion of surgically treated cases in Finland, which in turn contributes to the observed survival differences between Finland and other Nordic countries. Improving the outcomes of Finnish LC patients will require addressing key challenges such as delayed diagnostics and the low percentage of operatively treated patients.

## Supplementary Material



## Data Availability

According to Finnish laws and regulations, any data including details of personal information, cannot be publicly shared and only analyses where no person can be identified can be shared.
