# Supplementary material for: Outcome of lung cancer surgery and proportion of lung cancer patients eligible for surgery in five Finnish hospitals in 2018, real world study
Source: Acta Oncol. 2025 Jul 23;64:43398. doi: 10.2340/1651-226X.2025.43398 (PMC12305687; doi:10.2340/1651-226X.2025.43398)

Supplementary material has been published as submitted. It has not been copyedited, or typeset by Acta Oncologica

Supplementary materials

Table S1. Treatment of non-operated patients

Treatment of Non-Operated Patients

Treatment	N = 79[1]
**Chemotherapy**	10 / 79 (12.7%)
**Chemoradiotherapy**	13 / 79 (16.5%)
**Radiotherapy**	23 / 79 (29.1%)
Conventional RT	6 / 23 (26.1%)
SBRT	17 / 23 (73.9%)
**Immuno-oncologic and targeted drugs**	5 / 79 (6.3%)
**BSC**	28 / 79 (35.4%)

[1] n / N (%)

Table S2. 2- and 3-year OS rate of operated and non-operated patients according to stage

Overall Survival at 24 and 36 Months by Treatment Approach and Stage

Variable	N	24 Month (95% CI)	36 Month (95% CI)
**Operatively treated by stage**	77		
I		88% (74, 94)	79% (65, 88)
II		65% (40, 82)	45% (23, 65)
III		67% (28, 88)	67% (28, 88)
**Non-operatively treated by stage**	79		
I		57% (37, 72)	53% (34, 69)
II		20% (4.9, 42)	6.7% (0.4, 26)
III		21% (9.1, 35)	15% (5.4, 28)

Figure S1. Overall survival of non-operated patients according to stage

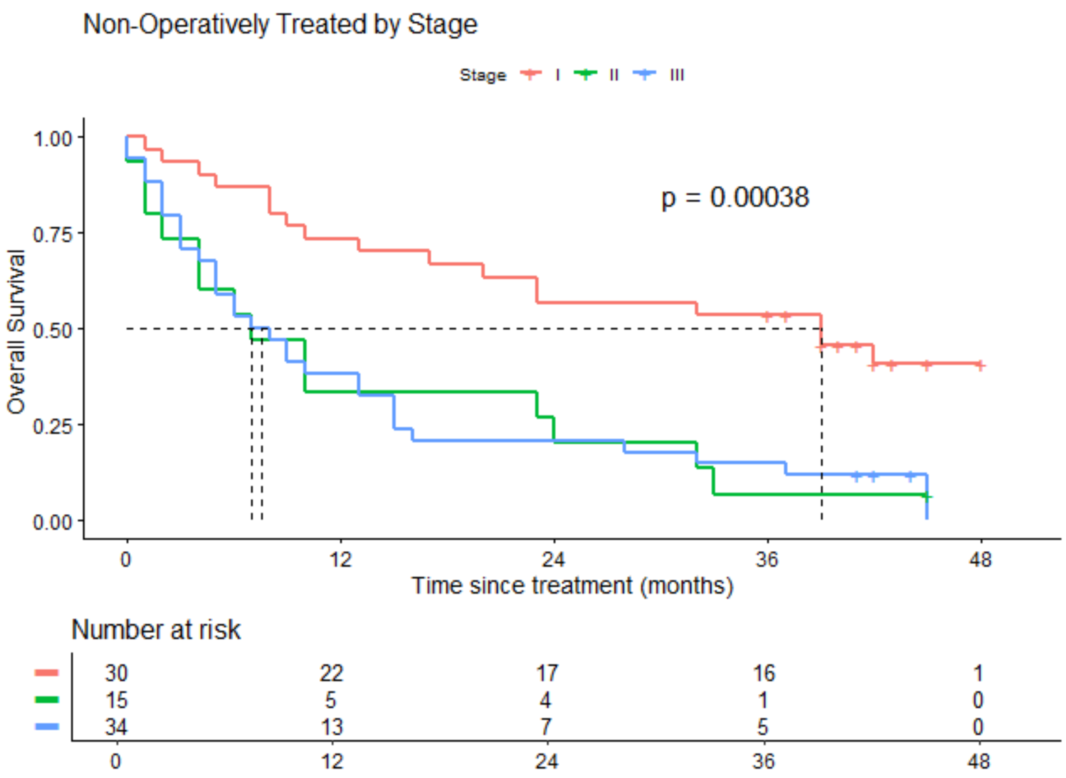

Figure S2. Overall survival of non-operated patients according to treatment

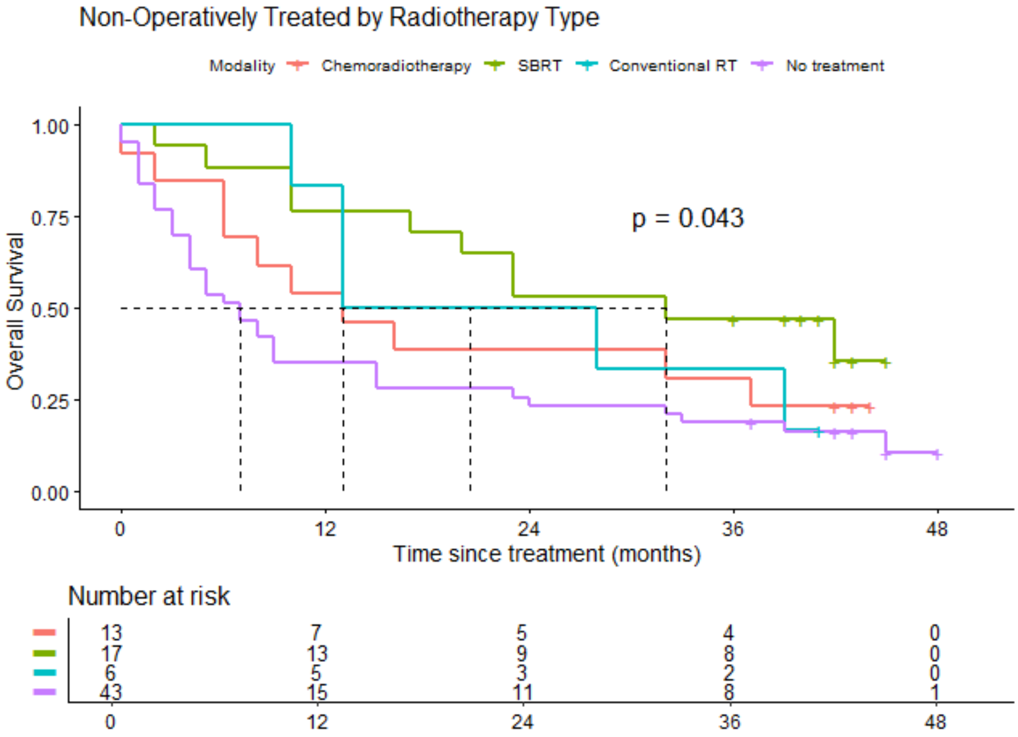

Supplement: Supplementary file 1 [file AO-64-43398-s1.pdf]
